# Pathogen Entrapment by Transglutaminase—A Conserved Early Innate Immune Mechanism

**DOI:** 10.1371/journal.ppat.1000763

**Published:** 2010-02-12

**Authors:** Zhi Wang, Christine Wilhelmsson, Pavel Hyrsl, Torsten G. Loof, Pavel Dobes, Martina Klupp, Olga Loseva, Matthias Mörgelin, Jennifer Iklé, Richard M. Cripps, Heiko Herwald, Ulrich Theopold

**Affiliations:** 1 Department of Molecular Biology and Functional Genomics, Stockholm University, Stockholm, Sweden; 2 Department of Animal Physiology and Immunology, Institute of Experimental Biology, Masaryk University, Brno, Czech Republic; 3 Department of Clinical Sciences, Lund University, Lund, Sweden; 4 Department of Genetics, Microbiology and Toxicology, Stockholm University, Stockholm, Sweden; 5 Department of Biology, University of New Mexico, Albuquerque, New Mexico, United States of America; Stanford University, United States of America

## Abstract

Clotting systems are required in almost all animals to prevent loss of body fluids after injury. Here, we show that despite the risks associated with its systemic activation, clotting is a hitherto little appreciated branch of the immune system. We compared clotting of human blood and insect hemolymph to study the best-conserved component of clotting systems, namely the *Drosophila* enzyme transglutaminase and its vertebrate homologue Factor XIIIa. Using labelled artificial substrates we observe that transglutaminase activity from both *Drosophila* hemolymph and human blood accumulates on microbial surfaces, leading to their sequestration into the clot. Using both a human and a natural insect pathogen we provide functional proof for an immune function for transglutaminase (TG). *Drosophila* larvae with reduced TG levels show increased mortality after septic injury. The same larvae are also more susceptible to a natural infection involving entomopathogenic nematodes and their symbiotic bacteria while neither phagocytosis, phenoloxidase or—as previously shown—the Toll or imd pathway contribute to immunity. These results firmly establish the hemolymph/blood clot as an important effector of early innate immunity, which helps to prevent septic infections. These findings will help to guide further strategies to reduce the damaging effects of clotting and enhance its beneficial contribution to immune reactions.

## Author Summary

One of the main functions of immune systems is to prevent the dissemination of microbes and the resulting sepsis. Blood clotting during sepsis has until now been primarily regarded as harmful, leading to the formation of widespread clots in blood vessels and as a result to organ failure. Here we show that clotting also has a protective function to limit and prevent infections. This is achieved by capturing bacteria in the clot. Our infection studies were performed in the insect model *Drosophila melanogaster* where, due to the presence of an open circulatory system, the negative effects of clotting are less pronounced. We show that clotting of hemolymph—the insect blood equivalent—is essential in *Drosophila* to prevent septic death arising from injection of bacteria or infection with a natural pathogen. We also show that both *Drosophila* transglutaminase and its human homologue clotting factor XIII are key enzymes for sequestration of bacteria in the clot matrix, indicating the conserved nature of the clot's function in immunity. Our data are expected to lead to a much stronger appreciation of the role of blood clotting in innate immunity, and will guide future therapies which target this process.

## Introduction

One of the major causes of organ failure during sepsis in humans is the systemic activation of coagulation which leads to the widespread deposition of fibrin deposits with the result of multiple organ failure due to reduced blood supply [1]. In contrast to these negative effects, it is less clear whether clotting also contributes to immunity in a positive way. The blood clot is ideally situated to prevent not only blood loss but also dissemination of infectious agents from the wound site [2] and has been proposed to have an immune-protective function during a very early stage of an infection [3]–[6]. Insects are injured frequently both by parasites such as nematodes [7] and parasitic wasps [8] as well as during copulation [9] and by predators increasing the risk of wound-borne systemic infections. Here we show that clotting has an important immune function by limiting the dissemination of infections. We focused on the enzyme transglutaminase/factor XIIIa, (TG and F XIII, respectively) which we studied both in the model insect *Drosophila melanogaster* and in humans. Chemically, TG and F XIII crosslink selected glutamines and lysines in proteins involved in clotting leading to ε-(γ-glutamyl)lysine bridges [10], which can be readily detected using artificial substrates. Phylogenetically, TG is the sole component of clotting cascades that has been conserved during evolution. Similarly in all species where coagulation has been studied, TG contributes to this process [11],[12]. Finally to our knowledge TG is present in the genome of all animals studied so far. This includes *Drosophila* where TG-activity can be detected in the clot and the enzyme contributes to clot formation [13],[14]. Like in other insects, coagulation of *Drosophila* hemolymph is based on an interaction between humoral and cellular procoagulants [15]. Humoral procoagulants in *Drosophila* comprise lipophorin, hexamerins, the hexamerin receptor (also called fat body protein 1, FPB1), the clotting factor fondue [5], and phenoloxidase, while hemolectin and tiggrin are derived from blood cells [16]. We hypothesized that *Drosophila* might be an ideal system to study the beneficial aspects of clotting since it has an open circulatory system in which obstruction of blood flow causes fewer problems than in vertebrates. We show that knockdown of *Drosophila* TG leads to increased mortality after injection of bacteria and in a natural infection model involving entomopathogenic nematodes and their associated bacteria. Both *Drosophila* hemolymph- and human blood clots sequester bacteria preventing their dissemination throughout the body. Our results firmly establish clotting as part of the innate immune system and relate it to other branches of immunity.

## Results

### *Drosophila* transglutaminase activity on microbial surfaces

**Figure 1:**
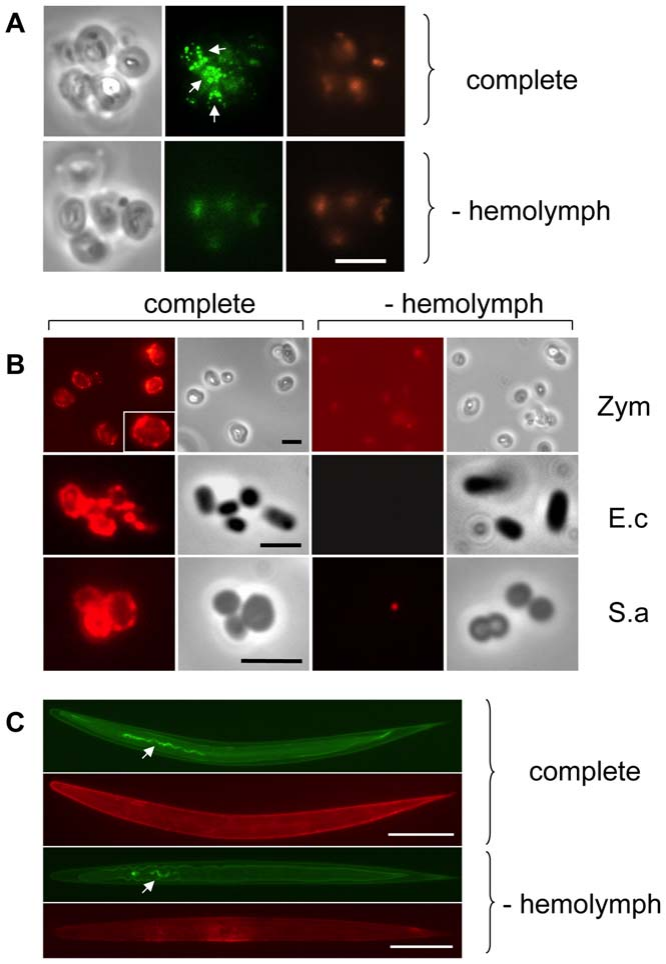
*Drosophila* Transglutaminase targets microbial surfaces. Microbes analyzed include: yeast zymosan particles (A,B: Zym), Gram− *E. coli* (E.c.) and Gram+ *S. aureus* (S.a.; B) and the entomopathogenic nematode *H. bacteriophora* (C); A: phase contrast exposure (left), immunocytochemistry with an antibody against ε-(γ-glutamyl)lysine bridges created by TG (middle) and autofluorescence in the red channel (right). Note the punctate deposits (arrows) on the zymosan particles which are absent in preparations that lack hemolymph. B-cad was used in B and C, showing TG-mediated incorporation of B-cad into Gln-containing protein substrates. The inset at the upper left in B is a twofold enlargement to show the punctate labelling. All exposures were analyzed using immunofluorescence detecting B-cad and the corresponding phase contrast exposures. Hemolymph was omitted as a control leading to a reduction of the signal for all microbes. The fluorescence exposure of the reaction with zymosan lacking hemolymph (Fig. 1B, right part) was 10× overexposed to underline the significant difference in labelling efficiency between the presence or absence of hemolymph. After omission of B-cad signals were undetectable in most cases (not shown). Scale bars in A and B correspond to 5µm. C: Nematodes (*Heterorhabditis bacteriophora*) were incubated with B-cad and hemolymph leading to the formation of aggregates on the cuticle (upper part). The lower part shows autofluorescence after omission of hemolymph. The presence of GFP-expressing *P. luminescens* is indicated by arrows. The scale bar corresponds to 100µm.

To investigate whether TG actively participates in the host response to infection, we challenged *Drosophila* hemolymph with microbes or microbe-derived immune elicitors, and then tested whether each treatment triggered activation of TG. For this purpose, *Drosophila* hemolymph was mixed with yeast cell wall preparations (zymosan beads), and the resulting aggregates probed with an antibody that recognizes ε-(γ-glutamyl)lysine bridges. Fluorescence microscopy of the aggregates revealed a punctate pattern mostly located at the interface between the particles (Fig. 1A). Such aggregates were also observed when hemolymph and zymosan were mixed in the presence of biotin-cadaverine (B-cad), a small primary amine capable of replacing lysine during TG-mediated crosslinking and which can serve to mark host proteins involved in crosslinking (Fig. 1B: Zym). Using the biotin tag, TG activity was also detectable on the surface of both DAP peptidoglycan-containing Gram− (*Escherichia coli*) and Lys peptidoglycan-containing Gram+ (*Staphylococcus aureus*) bacteria (Fig. 1B: E.c and S.a) and on the surface of entomopathogenic nematodes (Fig. 1C), which had been incubated with hemolymph. In all cases, the pattern after B-cad incorporation appeared to localize to small deposits on the microbial surfaces.

**Figure 2:**
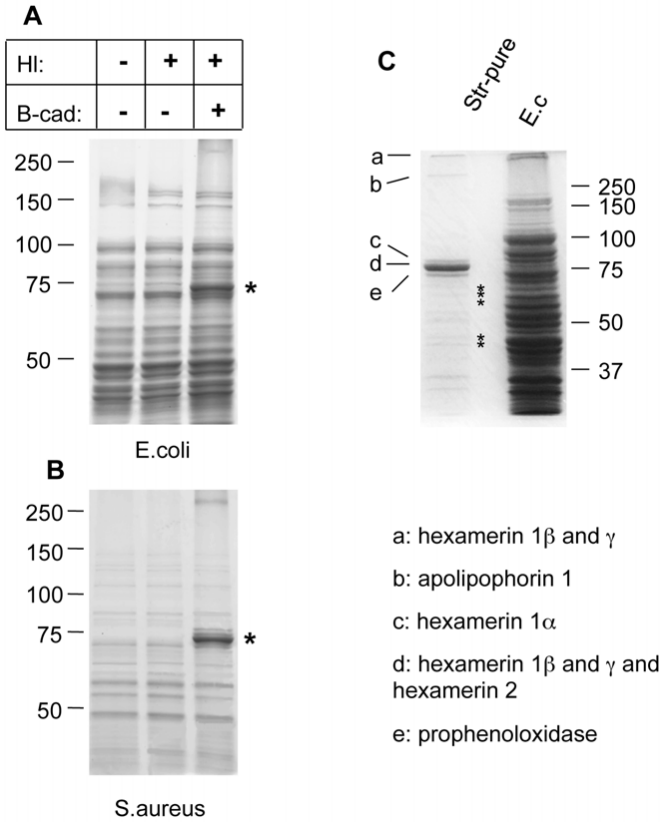
Humoral procoagulants bind to microbial surfaces. Lysates from *E. coli* (A) and *S. aureus* (B) were incubated in the presence of hemolymph (Hl), B-cad or the combination of both and analyzed using polyacrylamide gel electrophoresis. The additional band in the samples with Hl and B-cad (asterisk) represents hexamerin. Note that in the absence of B-cad hemolymph proteins form TG-crosslinked aggregates, thus preventing analysis with SDS-PAGE (see methods for further details). A similar pattern was obtained using *P. luminescens* (Figure S2). C: Proteins from an *E. coli* lysate treated like in Fig. 1 (right lane), were affinity-purified using streptavidin (Str-pure) and the identity of the purified proteins determined using mass spectrometry (E.c. shows a bacterial lysate, without hemolymph, the asterisks indicate breakdown products of hexamerin, see Table S1 for further details).

Analysis of both *E.coli* and *S.aureus* lysates after incubation with B-cad and hemolymph showed one prominent protein that had bound to bacteria and was identified as the humoral procoagulant hexamerin (Fig. 2A, B, see also Figure S1 for additional controls and binding to *Photorhabdus luminescens*). Using affinity purification of bacterial lysates after incubation with the biotinylated cadaverine, we confirmed hexamerin subunits as the major constituent of the aggregates, while less abundant protein components included phenoloxidase and lipophorin (Fig. 2C). These results show that upon septic injury, TG mediates the local formation of small aggregates on microbial surfaces, which incorporate humoral procoagulants. This is in line with our and others' earlier results, showing that bacteria and zymosan beads are sequestered by the clot ([4],[17] and Fig. S2).

### Factor XIII activity leads to sequestration of bacteria

**Figure 3:**
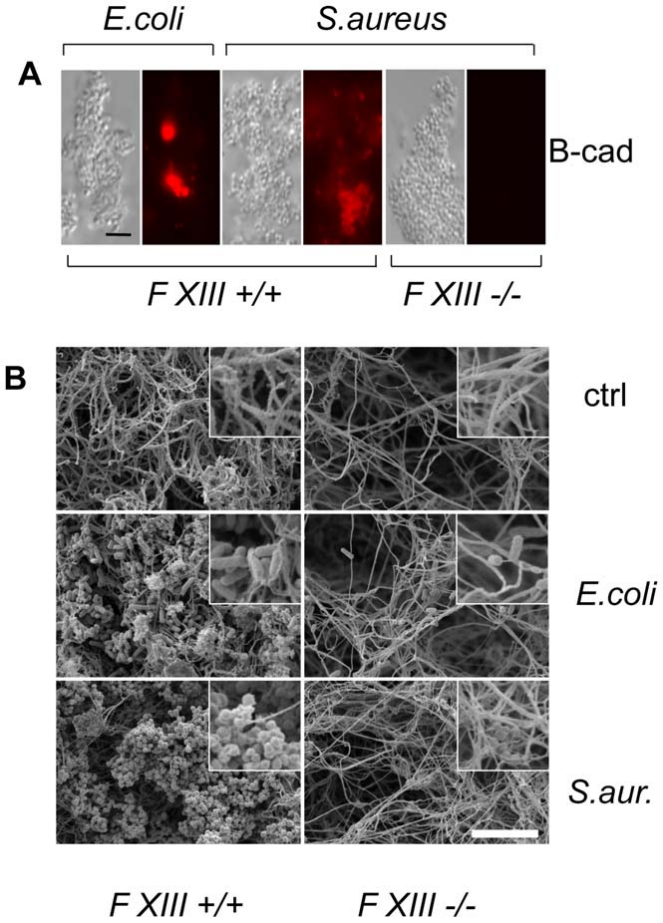
Human F XIII sequesters bacteria in the clot matrix. A: Plasma obtained from healthy donors (FXIII +/+) or donors with FXIII-deficiency (FXIII −/−) was activated with thrombin in the presence of *E. coli* or *S. aureus* (for details see Methods). B-cad was used to visualize FXIII-mediated incorporation at bacterial surfaces by immunofluorescence microscopy. Samples were also visualized by phase contrast to show the contour of the bacteria. B: SEM exposures of clots formed with normal plasma or FXIII-deficient plasma (the insets correspond to an 8-fold higher magnification). Clots were formed in the absence of bacteria (ctrl) or the presence of *E. coli* or *S. aureus* (scale bars correspond to 5 µm in A and 10 µm in B).

**Figure 4:**
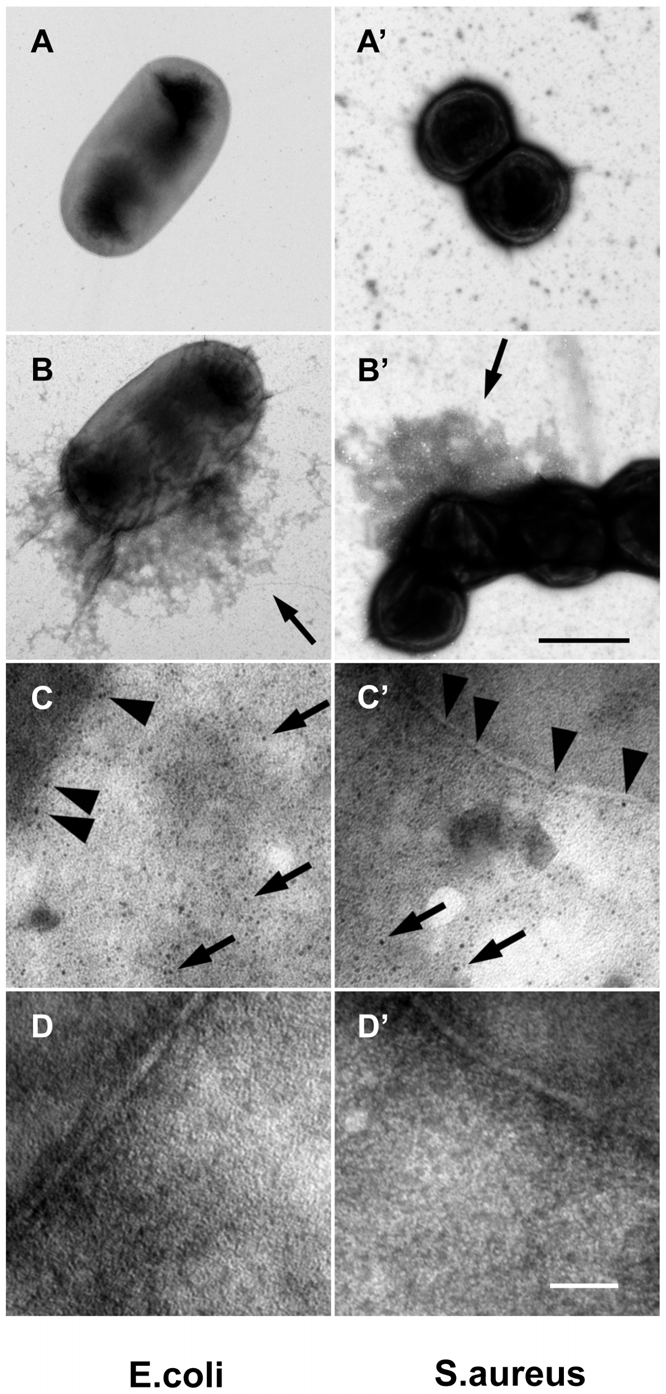
F XIII crosslinks are detectable on bacterial surfaces. *E. coli* (A–D) and *S. aureus* (A′–D′) bacteria were incubated with diluted and thrombin-activated normal (B–C′), F XIII-deficient plasma (D–D′) or left untreated (A–A′). Arrows in B and B′ point to plasma proteins crosslinked to the bacteria surface. Bacteria incubated with diluted and thrombin-activated normal (B–C′) or F XIII-deficient plasma (D–D′) were immunostained with a mouse anti-human gold-labeled ε-(γ-glutamyl) lysine-specific antibody. Arrowheads indicate crosslinking sites at the bacterial surface and arrows at crosslinking sites of crosslinked plasma proteins (scale bars correspond to 1 µm in B′ and 100 nm in D′). Please note that no colloidal gold staining was detected when bacteria were incubated with F XIII deficient plasma (D–D′).

To assess whether human F XIII has a role similar to *Drosophila* TG, we performed a parallel set of experiments by incubating B-cad with human plasma and either *E. coli* or *S. aureus*. In both samples, B-cad was deposited onto the bacterial surface albeit more efficiently with *S. aureus* as shown by fluorescence microscopy (Fig. 3A, left). No bacterial labelling was observed when F XIII-deficient plasma was used (Fig. 3A, right). This means that similar to *Drosophila* TG, human F XIII targets microbial surfaces. Subsequent scanning electron microscopy showed that the functional consequence of F XIII activity is the sequestration of bacteria by the clot matrix. Using normal plasma, both *S. aureus* and *E. coli* were efficiently immobilized (Fig. 3B left part). With both bacteria, sequestration was strongly reduced when F XIII-deficient plasma was used (Fig. 3B right part) or upon addition of monodansylcadaverine (MDC), a chemical inhibitor of TG with effects similar to B-cad ([14], Figure S3). Like in *Drosophila*, TG activity could also be detected on the surface of both *E. coli* and *S. aureus* using an antibody with specificity for TG crosslinks (Fig. 4). Altogether these data show that microbes are targeted by insect TG and human F XIII leading to their sequestration in the clot.

### Transglutaminase plays a role in immunity

**Figure 5:**
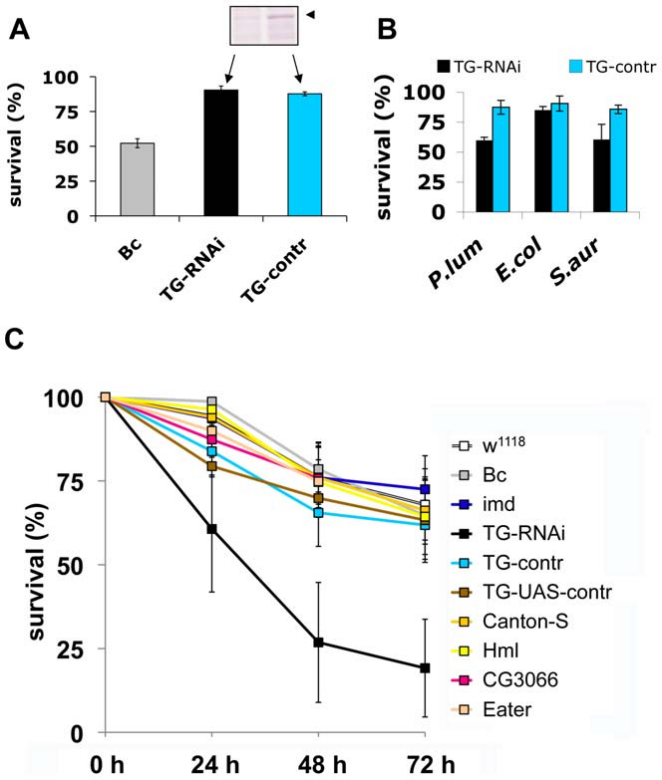
Larvae with reduced TG levels show immune defects. A: Lack of a wounding phenotype in TG knockdown lines. TG-knockdown larvae (*Act5C-Gal4>UAS-TG-RNAi*: labelled TG-RNAi); a control cross (*Act5C-Gal4>w^1118^*: labelled TG-control); and *Bc* larvae were injured and survival determined after 24 hours. The insert shows the reduction in TG protein levels (arrowhead) detected using TG-specific antibodies. B: TG knockdown larvae are more susceptible to some bacteria than control larvae. TG-RNAi larvae and control larvae were injected with *P. luminescens*, *E. coli* and *S. aureus* and survival scored after 24 h. C: TG-RNAi larvae are more susceptible to nematode infections. Larvae from the same strains like in (A) as well as the strain used for construction of knockdown lines (*w^1118^*); a homozygous *imd* mutant (*imd^Y47^*); a wildtype strain (Canton-S) Hml mutants (Hml); mutants lacking CG3066 and *eater* mutants were infected with *H. bacteriophora*. Mortality rates were determined at the indicated times post-infection. All data points in Figs. A–C represent at least triplicates (+/− s.d.), all experiments were performed at 22°C, see Methods for further details.

To test the functional requirement for TG activity in innate immunity, we used a previously described TG-RNAi line [14] with reduced expression of TG (inset in Fig. 5A) as well as a second independent TG knockdown line (see Methods). Aseptic injury of TG-RNAi larvae does not have a major effect upon survival, most likely due to the presence of redundant mechanisms [18]. In contrast, *black cells* (*Bc*) mutants lack phenoloxidase and show both poor clot formation [4] and strongly reduced viability upon wounding (Fig. 5A and [18],[19]). Therefore, any increased mortality that arises upon introduction of pathogens into TG-RNAi larvae is not expected to result from increased loss of hemolymph. To test whether TG has a function in immunity we next injected normal and TG knockdown *Drosophila* larvae with *E. coli*, *S. aureus* and the entomopathogenic bacterium *Photorhabdus luminescens*, the symbiotic bacterium of the nematode *Heterorhabditis bacteriophora* (Fig. 1C, arrows). While only a marginal non-significant effect on survival was observed using the non-pathogenic *E. coli*, both the human and insect pathogen led to increased mortality in TG-RNAi larvae (Fig. 4B). Thus it appeared that loss of TG led to a specific immune defect in *Drosophila* larvae. To test the suspected immune function more stringently, we used the entomopathogenic nematode *H. bacteriophora*, which offers several advantages: i) this nematode is a natural invasive insect pathogen, thus larvae are infected in a much more reproducible way than by any artificial injection [7]; ii) the infection includes induction of septicaemia due to the massive release of the nematode's symbiotic bacteria (*P. luminescens*), which are essential for the nematode's success as an entomopathogen [20] and which we already had found to be more infectious in TG-RNAi larvae (Fig. 5B); and iii) although both *Toll* and *imd* pathway-dependent antimicrobial peptides are induced after infection with *H. bacteriophora*, survival of larvae after nematode infection was unaffected by mutations in either of the two pathways [20]. This suggests that previously uncharacterized immune mechanisms are involved in surviving nematode infections. To test the involvement of cellular procoagulants, we included mutants lacking hemolectin [18]. To cover other immune reactions we included mutants in additional effector pathways: *Bc*, which lack active crystal cells; CG3066 mutants, which lack a protease required for prophenoloxidase activation [21] and mutants in the phagocytic receptor Eater [22].

**Figure 6:**
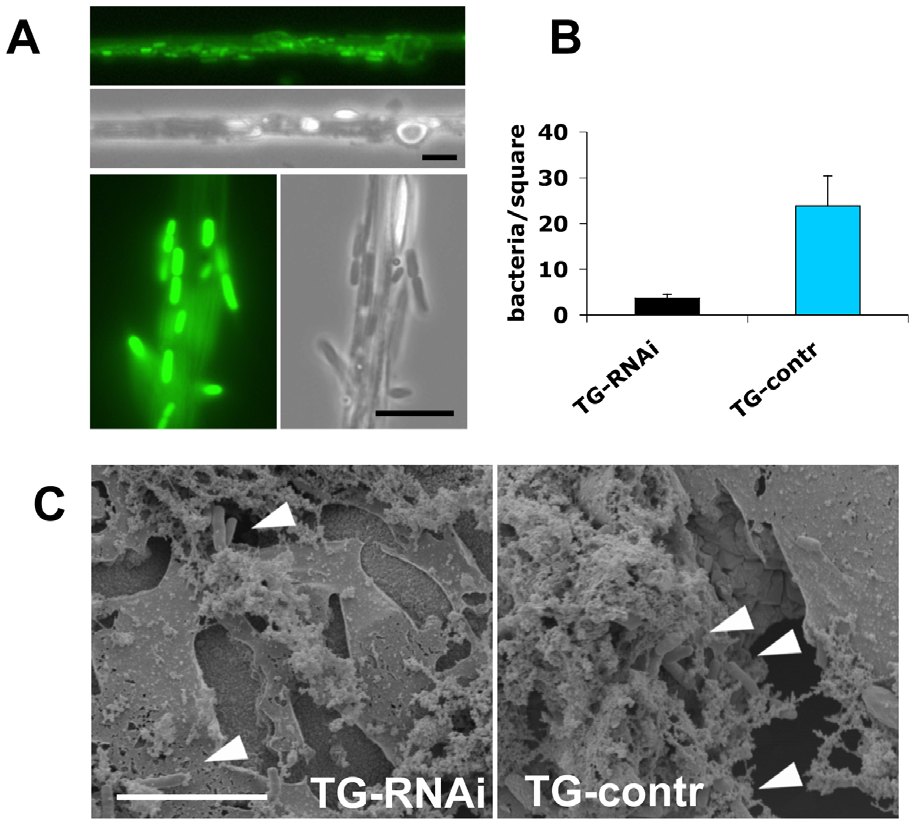
Clots from larvae with reduced TG levels sequester fewer bacteria. A: The clot from normal larvae captures *P. luminescens*. A clot bled from larvae expressing Fondue-FGP [14] was drawn out from hemolymph in the presence of GFP-expressing *P. luminescens* as described [16]. The clot is weakly labelled with Fondue-GFP [14]. The bacteria, which are immobilised in the clot [4] show a strong GFP signal (two sections are shown at different magnifications, the scale bars correspond to 10 µm). B: Hemolymph clots prepared as described [4] from larvae with less TG (TG-RNAi) and control larvae were captured and the number of sequestered bacteria determined under the microscope (P<0.01, performed in triplicates). C: Clots from both types of larvae were also analyzed using scanning electron microscopy (note the more brittle appearance of the clot from TG-RNAi larvae, which is also observed after addition of MDC: see [14]). The scale bar corresponds to 10 µm, the arrowheads indicate bacteria which have been incorporated into the clot.

Our experiments firstly confirm that *imd* mutant larvae have similar viability after infection with nematodes compared to control animals. In contrast, the TG-knockdown line used in Fig. 5A as well as the second TG RNAi line (see Methods) showed increased mortality, in line with a requirement for TG in immune function and survival after infection (Fig. 5C). Despite the wounding defects in *Bc* larvae, we found that *Bc* and CG3066 mutants showed normal viability after infection. We propose that, while phenoloxidase is critical to wound healing, it is less essential in the infection model we used here, most likely due to the production of a *P. luminescens* phenoloxidase inhibitor [23]. The cellular procoagulant hemolectin also appears dispensable for the response towards nematodes and their bacteria. Similarly, although we could confirm that lack of the phagocytic receptor eater reduces uptake of *P. luminescens* (Figure S4), this does not increase mortality indicating that phagoytosis too may be less critical for the defense towards *Heterorhabditis/Photorhabdus*. In contrast to *eater* mutants hemocytes from TG-RNAi larvae retained full phagocytic capacity (Figure S4). Further supporting the immune function of the clot, we found instead that *P. luminescens* is sequestered by the clot matrix (Fig. 6A). The clot's capacity to sequester bacteria is reduced in TG-RNAi larvae and the clot has a more brittle appearance in line with our previous results (Fig. 6B and C and [14]). Finally, the amount of hexamerin that binds to microbial surfaces is reduced in TG-RNAi larvae (Figure S5). Taken together these results firmly establish TG activity in the clot as an effective immune mechanism that plays a dominant role in infections such as with nematodes and their associated bacteria.

## Discussion

**Figure 7:**
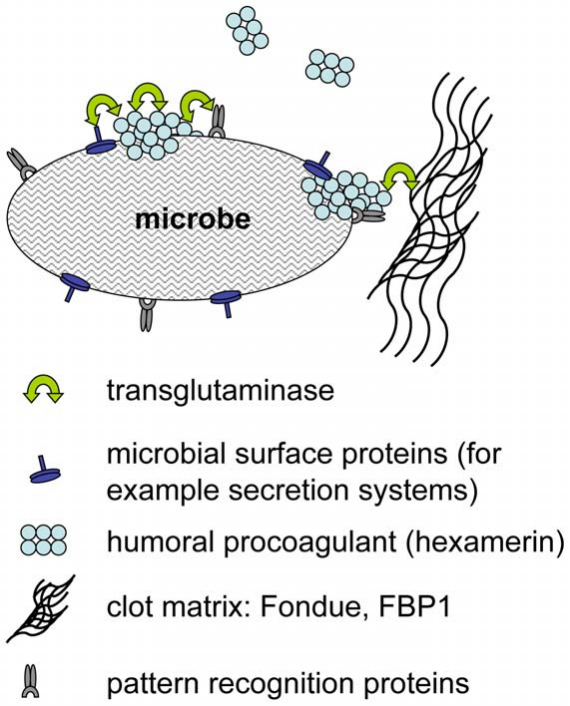
Hypothetical mechanisms for transglutaminase-mediated sequestration of microbes by the clot matrix. Transglutaminase crosslinks humoral procoagulants such as hexamerin and Fondue leading to their incorporation into the clot. Additional possible TG-substrates on microbes include microbial surface proteins such as secretion systems and recognition proteins with specificity for microbial patterns.

We have identified a previously underappreciated mechanism in the arsenal of insect and human innate immunity. Upon contact with hemolymph or blood, microbes are almost instantaneously targeted by TG activity leading to formation of small aggregates and ultimately to sequestration by the clot matrix (Fig. 7). Glutamine and lysine residues required for TG-crosslinking may potentially be present on different classes of proteins including: i) hemolymph proteins, such as hexamerin, assembled at the bacterial surface (see Fig. 2, of note hexamerins have been implied in immunity before [24],[25]); ii) bacterial proteins such as secretion systems or other virulence factors; or iii) host-derived recognition proteins with specificity for microbial patterns which have bound to the bacterial surface. Interestingly some hexamerins display lectin-like activity [26] and may act as recognition molecules in their own right. TG-substrates on microbes are subsequently linked to TG-substrates in the clot. We propose that in cooperation with phagocytosis, sequestration by the clot prevents dissemination of bacteria and systemic infections leading to a fast reduction in bacterial titres [6]. Alternatively the small aggregates we observe on microbial surfaces (Fig. 1) might play a role in immunity in their own right. Irrespective of the exact mechanism, TG- dependent activity appears to be the dominant immune mechanism during massive infiltration of bacteria such as after release from the nematode gut (Fig. 5C). In this case clot formation occurs in the absence of injury and is most likely identical to the formation of nodules to which it has been likened previously based on histological observations [27]. Future work will help to elucidate the exact route of *Photorhabdus* after their release into the hemolymph and whether TG contributes more to resistance or tolerance towards the bacteria [28],[29].

We observe that although TG activity can be detected on all microbial surfaces tested and targets the same hemolymph proteins on *E. coli*, *S. aureus* (Fig. 2A, B) and *P. luminescens* (Figure S3), TG knockdown lines show increased susceptibility to only some microbes (Fig. 3B). Further work will be required to show whether there are any qualitative differences between the aggregates that bind to different microbes and whether these explain the different efficacy of TG-mediated crosslinking. Regardless of the evolutionary variability of TG substrates in blood/hemolymph, TG itself is widely conserved and has been shown to contribute to clot formation in almost every species where clotting has been studied in any detail [15],[30]. For several animal models and for humans, evidence has been provided that the clot has a function in entrapping microbes [17],[31],[32]. Here we show for the first time its functional importance in a natural infection model. The mode in which TG contributes to immunity appears to be evolutionarily conserved providing yet another example for the successful use of insect models to decipher mechanisms that contribute to human immunity. TG activity has to be kept local in both *Drosophila* and humans but in contrast to insects with their open circulatory system systemic activation of TG in humans bears the additional risk of obstructing small blood vessels. Until now, focus on this negative aspect might have prevented full appreciation of the beneficial aspects of clotting. Our results fully agree with the observation that certain polymorphisms in clotting factors such as factor V Leiden which leads to a hypercoagulable state appear to be under balancing selection [33]. Epidemiological studies in humans and studies in animal models indicate that in addition to preventing bleeding more efficiently, Factor V Leiden might also protect from severe sepsis [34]. Future therapeutic strategies will thus have to aim at enhancing the helpful local effects of clotting while preventing its detrimental systemic effects. This appears even more vital in the light of the fact that we observe strong support for TG's immune function when using pathogenic bacteria such as *S. aureus* or *P. luminescens*.

## Methods

### Fly stocks

Flies were kept under standard conditions. The *Drosophila* strains included: a TG knockdown strain (Stock ID: 7356R-2, National Institute of Genetics Fly Stock Center, [14]). A second TG-RNAi strain produced independently with a different construct (Stock ID: 26101, Construct ID: 10774 from Vienna collection) showed similar reduced survival at all time points studied (24, 48 and 72 hours; p<0.05) although stronger effects were observed with 7356R-2 which was used for further studies. Crosses between TG-RNAi and *Act5C*-Gal4 show no morphological defects at 22°C and survive wounding equally well as control larvae (Fig. 4B); at higher temperatures, larvae appear normal although pupae displayed decreased eclosion rates. Additional strains include: *Canton-S*, *Black cells (Bc)*, and a P-element insertion mutant in CG3066 [28],[35] and *imd^Y47^*. Driver lines were *Act5C*-Gal4 and *ppl*-GAL4 (kindly provided by B. Lemaitre, Lausanne).

### Histochemistry with anti-crosslink antibody

Hemolymph from ten *w^1118^* larvae was incubated at a 50 fold dilution for five minutes at room temperature with *Drosophila* Ringer's solution containing the phenoloxidase inhibitor phenylthiourea (PTU) and Zymosan A (at a final concentration of 3×10^5^ beads/ml). The preparation was analyzed with the ε-(γ-glutamyl) lysine-specific antibody (at a dilution of 1∶100, Covalab mab0012). No signal was detected with secondary antibodies alone (not shown) or when hemolymph was omitted (Fig. 1A).

### Sequestration of bacteria by *Drosophila* clot

To analyze the sequestration of bacteria to fly clot, 10 larvae were bled into 2µl of bacterial suspension (*P.luminescens* expressing GFP) as described (hanging drop method, [4]). The clot was captured on an electron microscopy grid, washed 5 times with PBS and subsequently mounted on a new slide and the number of bacteria/square counted using fluorescence microscopy.

### Incorporation of B-cad into microbial surfaces

Ten *w^1118^* larvae were bled as described [14] followed by addition of either washed Zymosan A beads (SIGMA), or bacterial suspensions (*S. aureus* SH1000 or *E. coli* MG 1655, kind gifts from Håkan Steiner, Stockholm). After addition of biotincadaverine (Zedira) to 5 mM the preparation was incubated for 80 minutes at room temperature, centrifuged at 4000 g for 5 minutes, washed 3 times with *Drosophila* Ringer's solution and visualised using Streptavidine-Cy3 (SIGMA, note that due to competition with TG-mediated crosslinking, B-cad reduces aggregation of zymosan beads). Control preparations without biotincadaverine, which were prepared the same way as above showed labelling of just a few dead bacteria (see Fig. 1 for additional controls).

### Infection of *D. melanogaster* larvae with *H. bacteriophora*

Infection of *D. melanogaster* larvae with infective juveniles was modified according to Hallem et al. [20]. Infective juveniles from wildtype *H. bacteriophora* (H222, isolated from Pouzdřany, Czech Republic, kindly provided by Dr. Z. Mráček, Institute of Entomology, České Budějovice, Czech Republic) were collected after multiplying on *G. mellonella* larvae and used for infection according to [20] with the exception that the nematodes were applied using tissue paper at a multiplicity of 100 nematodes/larva. All experiments were performed at 22°C.

### TG antibody synthesis

Anti-TG guinea pig polyclonal antibody was produced by Invitrogen Corp. (Carlsbad, CA). Amino acid residues 757–773 were selected as the antigen (NH_2_-CQPNGSHRSSNIIRRRTD). The cysteine at the N-terminus is inserted to allow for conjugation with keyhole limpet hemocyanin. 50 µg of conjugate in Incomplete Freund's Adjuvant was injected into each of two guinea pigs at weeks 3, 5, and 8. Each guinea pig was “boosted” with 200 µg in Complete Freund's Adjuvant at week 10, and 100 µg in Incomplete Freund's Adjuvant at week 11. Exsanguination was carried out at week 21.

### Interactions of human F XIII with microbial surfaces in plasma

*S. aureus* SH1000 or *E. coli* MG 1655 bacteria were grown overnight in Todd-Hewitt-Broth or LB-Medium and washed 3× with sterile PBS. Human plasma obtained from healthy donors (purchased from the blood bank at Lund University Hospital, Lund, Sweden) or from donors with F XIII-deficiency (F XIII−/− plasma, purchased from George King BioMed Inc., Overland Park, KS, USA) was incubated with thrombin (Sigma, St. Louis, MO, USA) and bacteria. The peptide H-1998 (H-Gly-Pro-Arg-Pro-NH_2_) (Bachem, Bubendorf, Switzerland) was added to avoid clotting. Finally, biotincadaverine (Zedira, Darmstadt, Germany) was added to 5 mM and preparations were incubated for 1.5 h rotating at 37°C. After centrifugation at 8000 rpm and washing 3× with PBS streptavidin-Cy3 (Sigma, St. Louis, MO, USA) was added and the samples were incubated for 1 h rotating at room temperature. After 3× washing with PBS the preparations were mounted in glycerol and analyzed with a fluorescence microscope (Nikon, Tokyo, Japan) using a 100× objective. Control samples without biotincadaverine were prepared the same way as described above.

### Preparation of clots

Overnight cultures of *S. aureus* SH1000 or *E. coli* MG 1655 were grown in Todd-Hewitt-Broth or LB-Medium and washed 3× with sterile PBS. 50 µl of human plasma obtained from healthy donors or donors with F XIII-deficiency were incubated for 60 sec. at 37°C in a coagulometer (Amelung, Lemgo, Germany). 50 µl of bacterial solution were added followed by 60 sec. incubation at 37°C. Clotting was initiated by adding 100 µl of Hemoclot-Thrombin (Hyphen Bio-Med, Neuville-sur-Oise, France). Control clots without bacteria were generated by adding 100 µl of Hemoclot-Thrombin to 100 µl of human normal or F XIII−/− plasma. All clots were fixed in 2.5% glutaraldehyde in 0.15 M cacodylate buffer (pH 7.2) and analyzed by scanning electron microscopy. Samples were dehydrated with a graded series of ethanol, critical-point dried with CO_2_, and sputter coated with gold before examination in a JEOL JSM-350 scanning electron microscope (JEOL Ltd., Tokyo, Japan) operated at 5 kV accelerating voltage and a magnification of 2000. (as described elsewhere [36]). In some experiments the transglutaminase inhibitor monodansylcadaverine (MDC) (Sigma, St. Louis, MO, USA) was added to a final concentration of 5 mM to the plasma prior to the incubation with bacteria and the initiation of clotting.

### Negative staining

*E. coli* or *S. aureus* were grown overnight and 2×10^9^ bacteria per ml were incubated with human plasma obtained from healthy donors or patients with FXIII-deficiency. Plasma (diluted 1∶100 in 13 mM sodium citrate to avoid clotting) and bacteria were incubated for 30 Min at 37°C in the presence of thrombin and a mouse anti-human gold-labeled N ε gamma glutamyl Lysine [153-81D4] antibody (GeneTex Inc., Irvine, CA, USA), recognizing the crosslinking site of FXIII. Subsequently samples were adsorbed to 400 mesh carbon-coated copper grids for 1 minute, washed briefly with two drops of water, and stained with two drops of 0.75% uranyl formate. The grids were rendered hydrophilic by glow discharge at low pressure in air. Samples were observed in a Jeol 1200 EX transmission electron microscope operated at 60kV accelerating voltage as described earlier [37]. Control experiments were performed in the absence of bacteria and the antibody.

### Purification of proteins with a B-cad tag

Proteins containing a biotin tag were purified from sonicated bacterial lysates which had been treated as described above (see: Incorporation of B-cad into microbial surfaces) using streptavidin-containing magnetic beads (Dynal) according to the manufacturer's instruction except that *Drosophila* Ringer's solution was used for washes. Proteins were eluted using SDS-PAGE loading buffer and separated using PAGE.

### MALDI-TOF mass spectrometry analysis and protein identification

After affinity purification on streptavidin proteins (see Fig. 2C) were identified as described [16]. The hexamerin bands in Figs. 2A and B were in sufficient amounts to identify them without further purification. The results of the complete identification are summarised in Table S1.

### Statistical analysis

Samples from 5 infection experiments using *w^1118^* were initially tested positive for normality (Lilliefors test). Strain mortality was subsequently compared using ANOVA followed by Tukey's test for significance. The results were confirmed using a log-Rank test on survival curves.

## Supporting Information

Figure S1 Humoral procoagulants bind to *E. coli* (A) and *P. luminescens* (B) surfaces. Bacterial lysates were incubated in the presence of hemolymph (Hl), B-cad or the combination of both or with B-cad alone (in the case of *E. coli*) and analyzed using polyacrylamide gel electrophoresis. The additional band in the samples with Hl and B-cad (asterisks) represents hexamerin. Note that in the absence of B-cad hemolymph proteins form TG-crosslinked aggregates, thus preventing analysis with SDS-PAGE (see methods for further details).
(0.53 MB TIF)

Figure S2 Zymosan particles are sequestered by the clot matrix. A drawout (A and [16] was performed in the presence of zymosan and the resulting fibers analyzed under fluorescence microscopy (B) and phase contrast (C). Zymosan beads visible due to autofluorescence are indicated by arrowheads, fat body debris released during wounding is also incorporated (*).
(1.27 MB TIF)

Figure S3 Sequestration of bacteria is inhibited by the TG inhibitor monodansylcadaverine (MDC). Clots were prepared as described (see Fig. 3B) in the presence and absence of MDC alone or in the presence of either *E. coli* or *S. aureus* SH1000. The scale bar corresponds to 10 µm.
(3.75 MB TIF)

Figure S4 Hemocytes from eater mutants but not from TG-RNAi larvae show reduced phagocytosis of *P. luminescens*. The percentage of hemocytes that had taken up bacteria was counted essentially as described [22] after mixing with GFP-expressing *P. luminescens* and incubation for 30 minutes. Note that in contrast to eater mutants (p = 8.1×10^−8^ compared to controls: TG-ctrl), hemocytes from TG-RNAi lines show normal phagocytic capacity.
(0.11 MB TIF)

Figure S5 Hexamerin binding to microbes is reduced in TG-RNAi larvae. Proteins binding to zymosan in the presence of biotincadaverine were analyzed using polyacrylamide-gelelectrophoresis. Both complete zymosan beads (−) as well as beads after pullout [5] on peanut agglutinin (PNA, +) are shown for a control cross (TG-contr) and a TG-knockdown (TG-RNAi). Note that the amount of hexamerin is reduced after TG-RNAi for both treatments.
(0.31 MB TIF)

Table S1 Identification of microaggregate proteins.
(0.05 MB DOC)
