# Supplementary figures and images for: Pathogen Entrapment by Transglutaminase—A Conserved Early Innate Immune Mechanism
Source: PLoS Pathog. 2010 Feb 12;6(2):e1000763. doi: 10.1371/journal.ppat.1000763 (PMC2820530; doi:10.1371/journal.ppat.1000763)

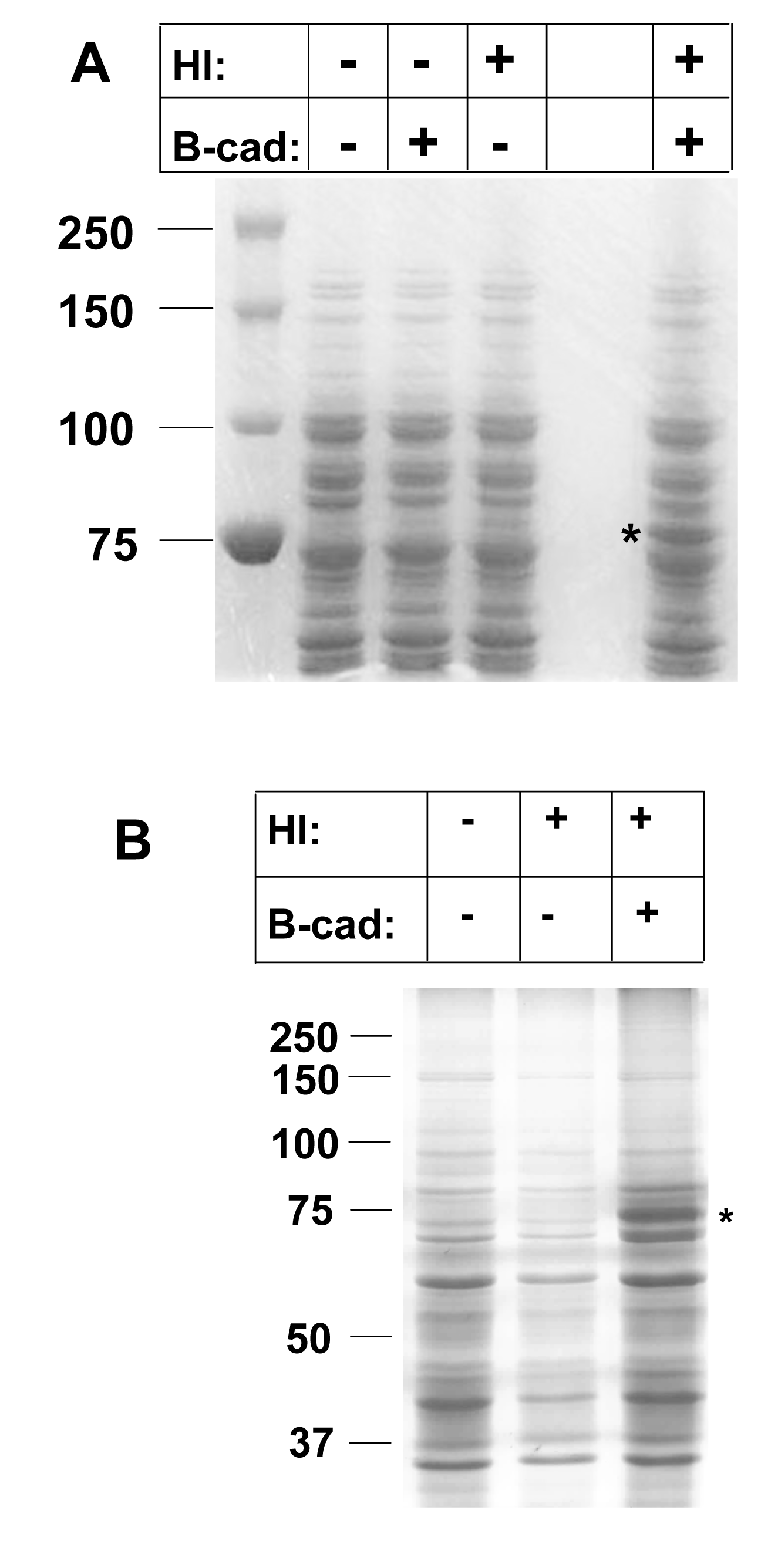

Supplement: Figure S1 — Humoral procoagulants bind to E. coli (A) and P. luminescens (B) surfaces. Bacterial lysates were incubated in the presence of hemolymph (Hl), B-cad or the combination of both or with B-cad alone (in the case of E. coli) and analyzed using polyacrylamide gel electrophoresis. The additional band in the samples with Hl and B-cad (asterisks) represents hexamerin. Note that in the absence of B-cad hemolymph proteins form TG-crosslinked aggregates, thus preventing analysis with SDS-PAGE (see methods for further details). (0.53 MB TIF) [file ppat.1000763.s001.tif]

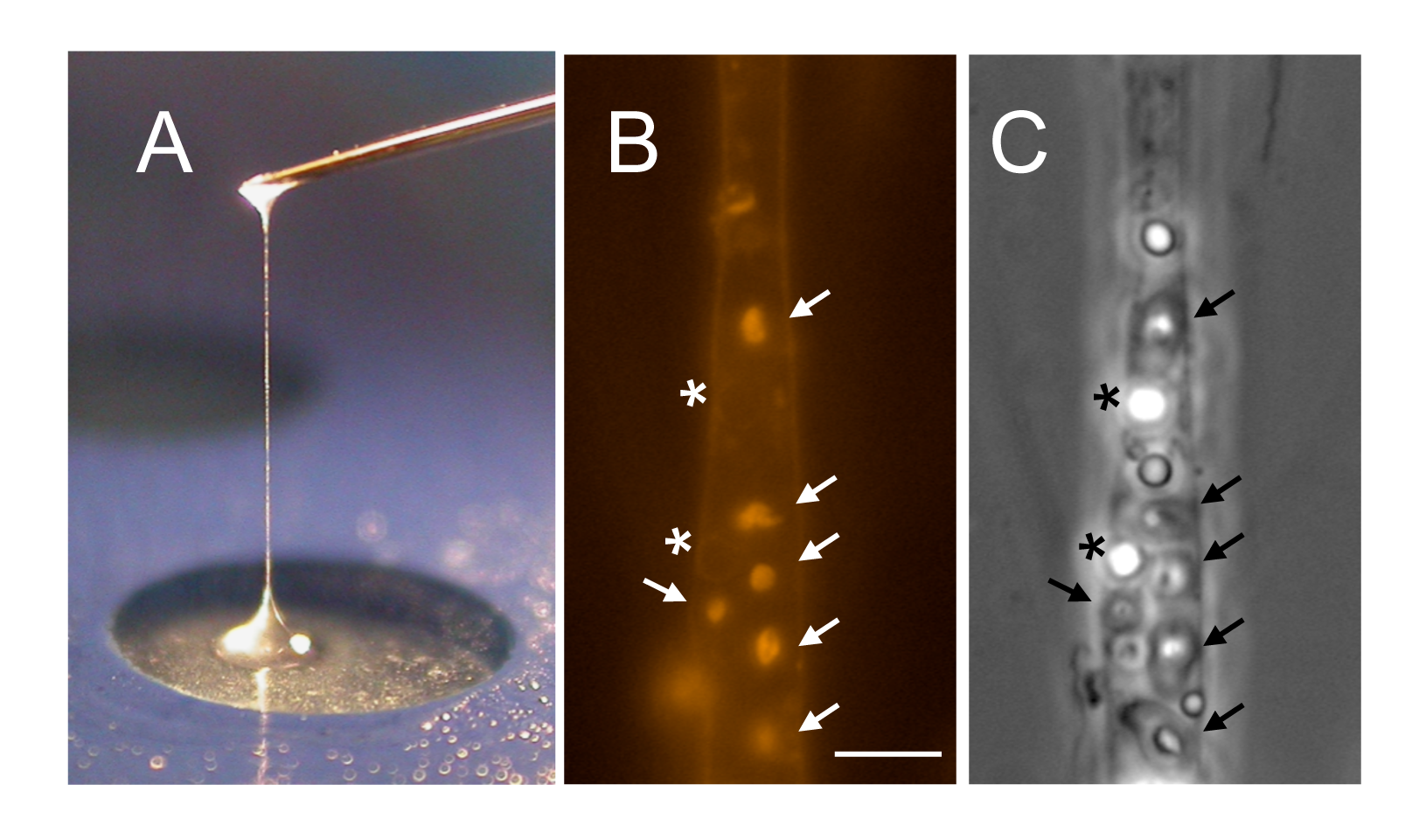

Supplement: Figure S2 — Zymosan particles are sequestered by the clot matrix. A drawout (A and [16] was performed in the presence of zymosan and the resulting fibers analyzed under fluorescence microscopy (B) and phase contrast (C). Zymosan beads visible due to autofluorescence are indicated by arrowheads, fat body debris released during wounding is also incorporated (*). (1.27 MB TIF) [file ppat.1000763.s002.tif]

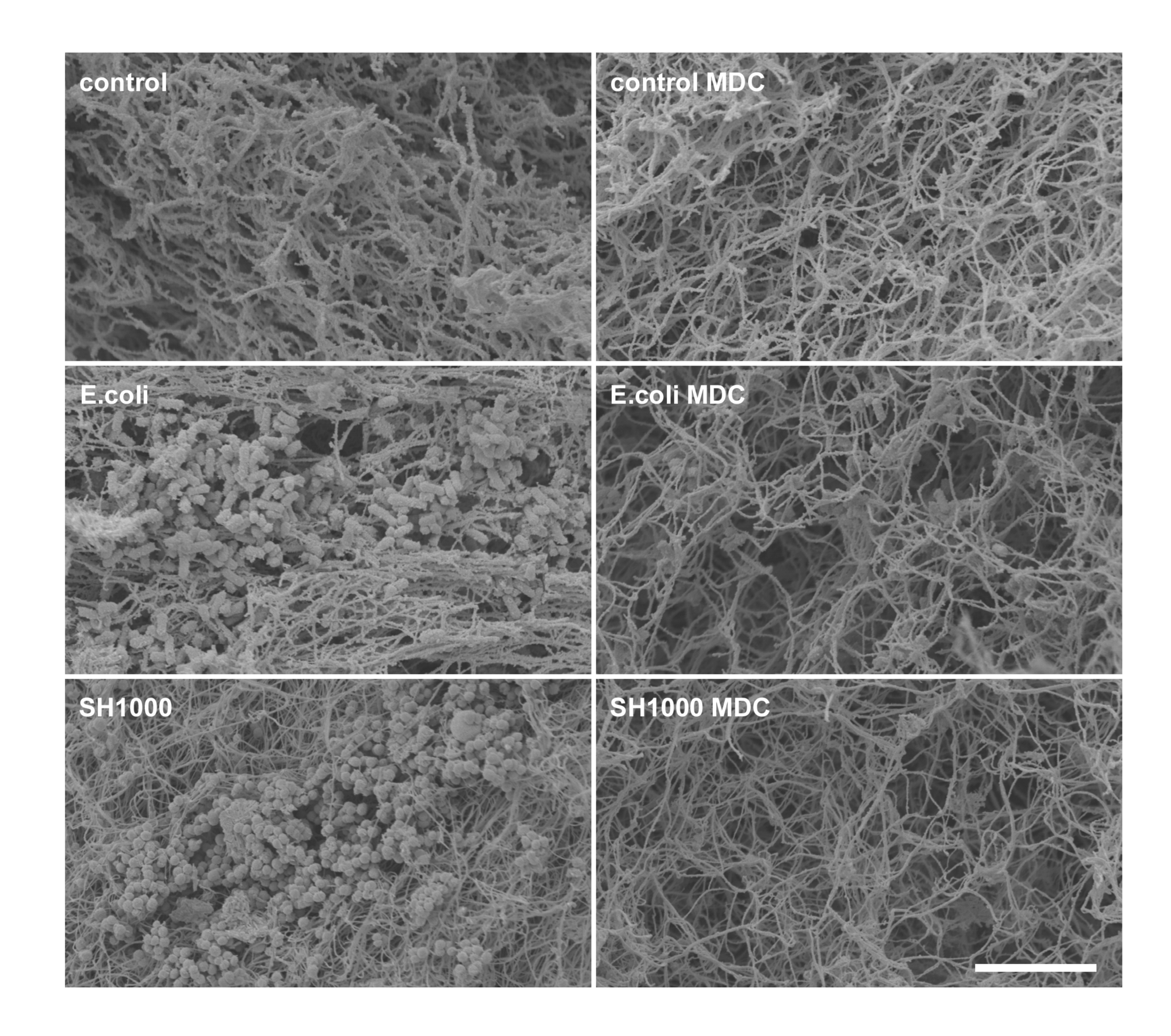

Supplement: Figure S3 — Sequestration of bacteria is inhibited by the TG inhibitor monodansylcadaverine (MDC). Clots were prepared as described (see Fig. 3B) in the presence and absence of MDC alone or in the presence of either E. coli or S. aureus SH1000. The scale bar corresponds to 10 µm. (3.75 MB TIF) [file ppat.1000763.s003.tif]

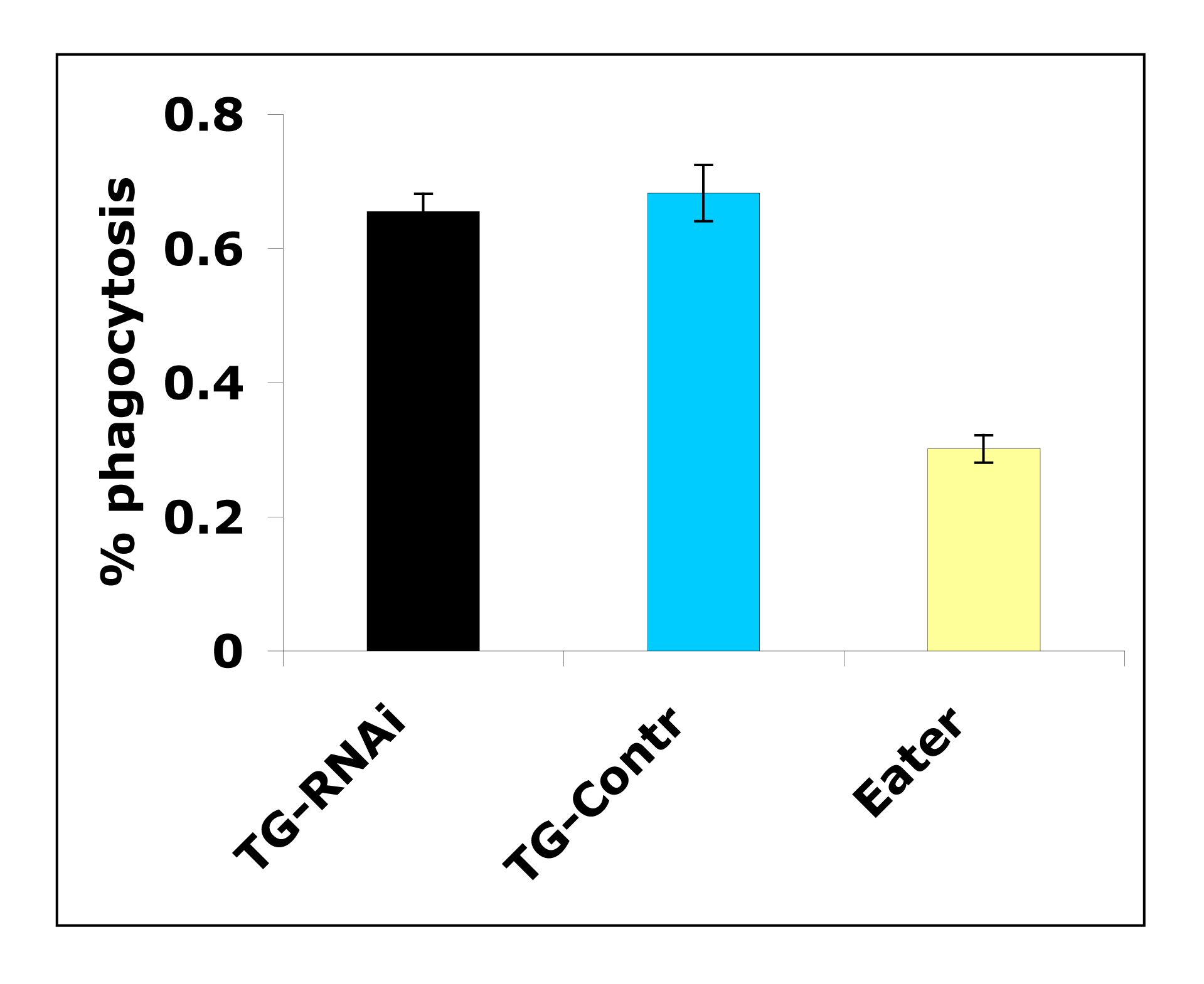

Supplement: Figure S4 — Hemocytes from eater mutants but not from TG-RNAi larvae show reduced phagocytosis of P. luminescens. The percentage of hemocytes that had taken up bacteria was counted essentially as described [22] after mixing with GFP-expressing P. luminescens and incubation for 30 minutes. Note that in contrast to eater mutants (p = 8.1×10^−8^ compared to controls: TG-ctrl), hemocytes from TG-RNAi lines show normal phagocytic capacity. (0.11 MB TIF) [file ppat.1000763.s004.tif]

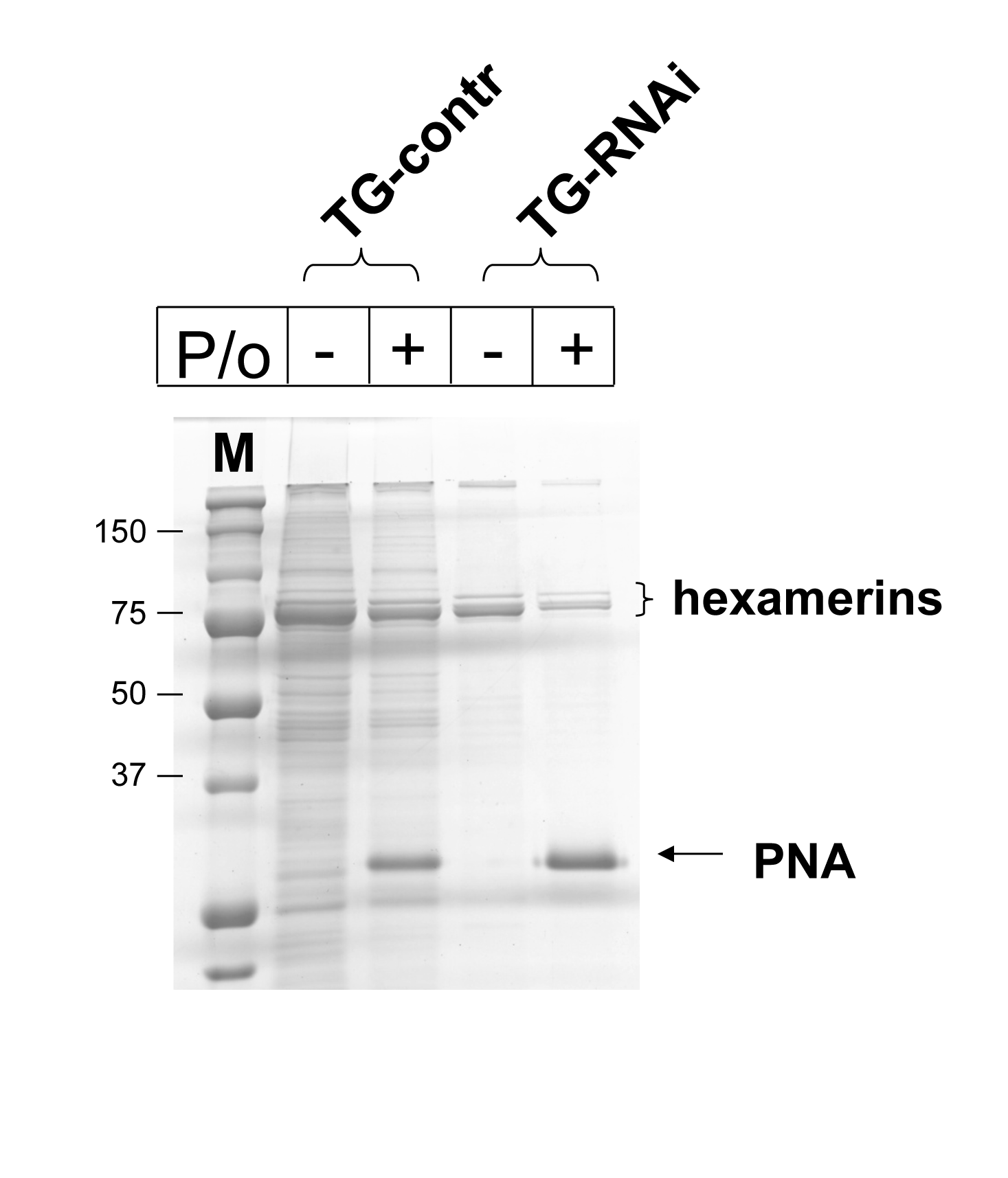

Supplement: Figure S5 — Hexamerin binding to microbes is reduced in TG-RNAi larvae. Proteins binding to zymosan in the presence of biotincadaverine were analyzed using polyacrylamide-gelelectrophoresis. Both complete zymosan beads (−) as well as beads after pullout [5] on peanut agglutinin (PNA, +) are shown for a control cross (TG-contr) and a TG-knockdown (TG-RNAi). Note that the amount of hexamerin is reduced after TG-RNAi for both treatments. (0.31 MB TIF) [file ppat.1000763.s005.tif]
