# Supplementary material for: Pathogen Entrapment by Transglutaminase—A Conserved Early Innate Immune Mechanism
Source: PLoS Pathog. 2010 Feb 12;6(2):e1000763. doi: 10.1371/journal.ppat.1000763 (PMC2820530; doi:10.1371/journal.ppat.1000763)
Supplement: Table S1 — Identification of microaggregate proteins. (0.05 MB DOC) [file ppat.1000763.s006.doc]

| **Band no.** | **Gene identity**  **(UniProtKB ID)** | **Protein name**  **(gene name)** | **Theoretical** | | **Sequence coverage, %** |
| --- | --- | --- | --- | --- | --- |
|  |  |  | **p*I*** | **M_r_ kDa** |  |
| 1 | **CG4178**  (P11996)  **CG6821**  (P11997) | **Larval serum protein 1 beta chain** 1 (**Lsp1b**)  **Larval serum protein 1 gamma chain** (Lsp1-g) | 5.4  5.3 | 95.9  93.4 | 20  12 |
| 2 | **CG11064**  (Q9V496) | Apolipophorin-1 (Rfabg) | 7.0 | 295.0 | 16 |
| 3 | **CG8542**  (P11995) | **Larval serum protein 1 alpha chain (Lsp1a)** | 5.72 | 99.0 | 26 |
| 4 | **CG6821**  (P11997)  **CG4178**  (P11996)  **CG6806**  (Q24388) | **Larval serum protein 1 gamma chain** (Lsp1-g)  **Larval serum protein 1 beta chain** 1-(**Lsp1b**)  Larval serum protein 2  (Lsp2) | 5.38  5.4  5.9 | 93.4  95.9  83.3 | 12  10  11 |
| 5 | **CG8193**  (Q9V521)  **CG5779**  (Q7K2W6) | Phenol oxidase  Prophenol oxidase (Bc) | 6.5  6.1 | 79.5  79.4 | 30  30 |

**Supplemental Table 1:** Identification of microaggregate proteins. Note that the theoretical M_r_ includes signal peptides (note that larval serum protein is synonymous with hexamerin)**.**
